# Anxiolytic-Like and Antinociceptive Effects of 2(*S*)-Neoponcirin in Mice

**DOI:** 10.3390/molecules18077584

**Published:** 2013-06-28

**Authors:** Julia Cassani, Anna G. Escalona Araujo, Mariano Martínez-Vázquez, Norberto Manjarrez, Julia Moreno, Rosa Estrada-Reyes

**Affiliations:** 1 Departamento de Sistemas Biológicos, Universidad Autónoma Metropolitana Unidad Xochimilco, Mexico D.F. C.P. 04960, Mexico; 2 Laboratorio de Fitofarmacología, Dirección de Investigaciones en Neurociencias, Instituto Nacional de Psiquiatría Ramón de la Fuente Muñiz, Calzada Mexico-Xochimilco 101, Col. San Lorenzo Huipulco, Delegación Tlalpan, Mexico D.F, 14370, Mexico; 3 Instituto de Química, Universidad Nacional Autónoma de Mexico, Coyoacan, Mexico D.F. 04510, Mexico

**Keywords:** neoponcirin, anxiolytic-like, *Clinopodium mexicanum*, toronjil de monte, analgesic, GABAergic system

## Abstract

*Study aims*: 2(*S*)-neopincirin (NEO) is a constituent from of *Clinopodium mexicanum*, which is used in traditional Mexican herbal medicine for its tranquilizing and analgesic properties. This study investigated the anxiolytic-like, sedative and antinociceptive effects of NEO in several mice models. *Material and methods*: The anxiolytic-like effect was evaluated in the hole-board (HBT) and Open Field Tests (OFT); sedative effect was evaluated in sleeping time induced by sodium pentobarbital, and its antinociceptive actions were measured in the hot plate test. To evaluate if the GABA receptor could be involved in the anxiolytic-like effect produced by NEO, in independent experiments, the effects produced by co-administration of NEO *plus* muscimol (MUS) and NEO *plus* Pitrotoxin (PTX) were evaluated in the HBT. *Results:* NEO was isolated from *Clinopodium mexicanum* leaves. The NMR, MS and optic rotation data helped establish its identity as (*2S*)-5-hydroxy-4′-methoxyflavanone-7-*O*-{*β*-glucopyranosyl-(1→6)-*β*-rhamnoside}. NEO showed an anxiolytic-like effect and was able to counter the nociception induced by a thermal stimulus in a dose-dependent manner. PTX blocked the anxiolytic-like effect of NEO, while MUS was able to enhance it. *Conclusions:* The findings of present work demonstrated that NEO possesses anxiolytic-like and antinociceptive effects in mice. Such effects are not associated with changes in the locomotor activity. These results supported the notion that anxiolytic-like effect of NEO involves the participation of GABAergic system.

## 1. Introduction

Flavonoids comprise a class of important secondary metabolites in vascular plants, vegetables, seeds, herbs, spices, flowers, as well as tea and red wine, and citrus fruits. More than 6,000 different flavonoids have been described and the properties of many of them studied. Flavonoids are low-molecular-weight, phenylbenzopyrones (phenylchromones) with an assortment of structures based on a common three-ring nucleus. Pharmacological properties of flavonoids cover a wide spectrum of actions such as inhibition of many enzymes, for example protein kinase C, phospholipase A_2_, Na+ and K^+^-ATP-ases, lipooxygenases and cyclooxygenases, inhibit HIV-1 proteinase, among others, and anti-inflammatory, antitoxic, antiallergic, anticarcinogenic, antiviral, and cytotoxic antitumoral properties [1,2,3]. Perhaps its most studied properties have been their anti-oxidant effects, which reflect their capacity to protect the cells from death produced by oxidative stress, implicated in Ca^2+^-induced neuroexcitotoxicity and several pathologies such as Alzheimer’s and Parkinson’s diseases [4,5,6]. The sedative effect of some glycoside flavonoids has been reported [7,8]. However, until details about their anxiolytic effects are scanty.

Previously, we reported the antinociceptive and sedative effects of an aqueous extract of *Clinopodium mexicanum* Benth Govaerts (Lamiaceae), known as “toronjil de monte” (hill’s hyssop), a medicinal plant used as a tranquilizer in Mexican traditional medicine [9]. Neoponcirin (syn. Isosakurenetin-7-*O*-rutinoside and didymin) is one of the major constituents of a complex mixture of flavone and flavanone glycosides present in the aqueous and methanol extracts of this medicinal plant [9].

On the other hand, with respect to the molecular structure of flavanone glycosides the following points are worthy of consideration:

(1) The flavanone aglycone system has an asymmetric carbon in the position two, which can be in the *S* or *R* orientation. Though all natural flavanones have a thermodynamically favored conformation with the C-2 aryl group in an equatorial position, and this implies that all levorotatory flavanones possess a 2*S* configuration, the racemization of products (affording *S*/*R* mixtures) usually takes place during the isolation process. Thus, it is a fact that the *S* or *R* diasteroisomer has different chemical and physical properties, and thereby potentially different pharmacological actions.
(2) The binding between sugar residues can take place in two different positions; 1→2 (glc→rha) or 1→6 (glc→rha), producing two regioisomers. Likewise, the impact of regioisomers in the pharmacological properties of drugs is well known, for example: hesperidin (1→2; glc→rha) [10] of bitter taste produces sedative effects that does not involve the GABAergic system, While, sweet tasting neohesperidin (1→6; glc→rha) of is a GABAergic modulator, without sedative effects *per se*.

Considering the above, the aim of the present work was to evaluate in several mice behavioral paradigms the sedative, anxiolytic-like and antinociceptive effects of the epimer (*2S*)-5-hydroxy-4′methoxy-flavanone-7-*O*-{*β*-glucopyranosyl-(1→6)-*β*-rhamnoside} (NEO) isolated from leaves of *Clinopodium mexicanum* Benth Govaerts (Lamiaceae).

Anxiolytic-like and sedative effects of NEO were compared with those produced by diazepam (DZ), an anxiolytic and sedative drug commonly used in clinical practice. Furthermore, the effects of picrotoxin (PTX); an antagonist GABAergic and the agonist muscimol (MUS) on the anxiolytic-like effects of NEO were analyzed. NEO was isolated and purified from a methanol crude extract of *C. mexicanum* by chromatography and recrystallization methods. The purity of the isolated compound was determined by HPLC analysis and its structure was elucidated by proton ^1^H-NMR, ^13^C-NMR, High Performance Liquid Chromatography-Electro Spray Ions-Mass Spectrometry and optical rotation data.

## 2. Results and Discussion

*Clinopodium mexicanum* Benth Govaerts (Lamiaceae) is a plant used in traditional medicine in Central Mexico for its analgesic and tranquilizing properties. Previously we reported the sedative and antinociceptive effects of an aqueous extract of *C. mexicanum* leaves. The occurrence of neoponcirin, a flavanone rutinoside, was described in that paper as one of the major constituents of a complex mixture obtained from methanol and aqueous extracts of *C. mexicanum* [9].

On the other hand, flavonoid compounds exert several effects on the Central Nervous System (CNS), the sedative and depressant effects of some flavanone glycosides, such as linarin, naringin, and hesperidin have extensively been described in particular [10,11,12]. As we mentioned above, the antianxiety and antinociceptive effects of those compounds have been scarcely explored.

Thus, we decided to evaluate the anxiolytic-like, sedative and antinociceptive effects of the 2*S* epimer of neoponcirin (NEO) in several mice behavioral models. To achieve this aim the isolation and structural characterization of the *S* epimer of neoponcirin was carried out.

### 2.1.Isolation and Identification of 2S-Neoponcirin

**Figure 1:**
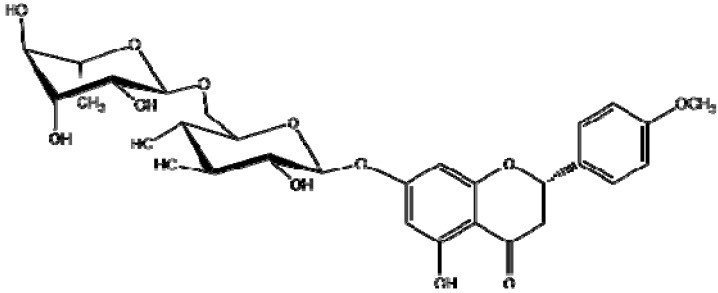
Structure of NEO.

NEO was isolated from a complex mixture obtained starting from the MeOH extract of *C. mexicanum*, as a white amorphous solid, melting point = 256–258 °C, which MS was consistent with a molecular formula of C_28_H_34_O_14_ that requires [M^+^] at *m/z* = 594 (positive ESI-MS *m/z* 595 (M + H)^+^ and ${\left[\alpha \right]}_{D}^{25}$ = −45°. The ^13^C-NMR spectrum exhibits signals of 28 carbon atoms, including one methoxyl carbon, one methyl, two methylenes, seventeen methines and seven quaternary carbons, which were assigned with the aid of Distortionless Enhancement by Polarization Transfer (DEPT), Heteronuclear Single-Quantum Correlation (HSQC) and Heteronuclear Multiple-Bond Correlation (HMBC) experiments. In the NMR spectra typical signal systems for the A and B rings of flavonoid compounds were observed. The proton spectrum showed a set of signals of an AA’BB’ system at δ 7.45 and 6.98 ppm corresponding to the H-2′, 6′ and H-3′, 5′ protons of the ring B. The assignment was further confirmed by the ^13^C-NMR spectrum (128 for C-2′/C-6′ and 113 for C-3′/C-5′, other characteristic signals are that showed the presence of meta coupled signals at δ 6.12 ppm and these were assigned to H-6 and H-8 of the ring A respectively, corresponding to C-6 at 96.5 and C-8 at 95.5 ppm. These observations suggested that the C-5 and C-7 positions were oxygenated, the substitution of C-5 was confirmed by the presence of a double signal at 4.98 ppm in the ^13^C-NMR spectrum whose coupling constant (*J* = 7.5 Hz) corresponds to one proton of a glucose in the *β* position. The most remarkable signal is the one that shows the interaction between the H_2_ and H_3_ (ax) with a long coupling constant of *J*_2,3_ = 12.6 Hz that indicates that configuration of C-2 aryl group of heterocyclic ring is equatorial, which implies that the molecule has a 2*S* configuration, as shown in Figure 1. The spectra data all together thus helped to establish its structure as (*2S*)-5-hydroxy-4′methoxyflavanone-7-*O*-{*β*-gluco-pyranosyl-(1→6)-*β*-rhamnoside).

**Figure 2:**
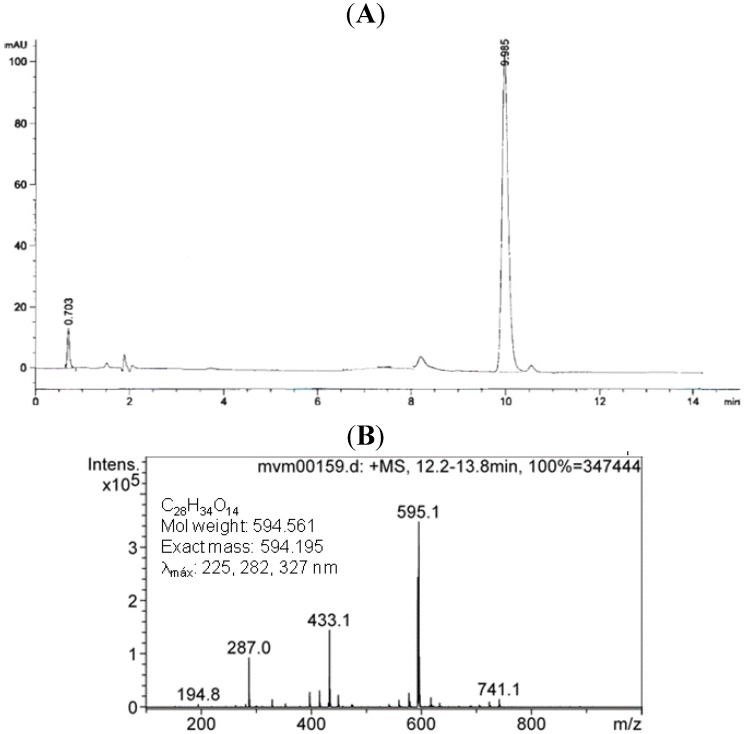
Chromatogram (**A**) and mass spectrum of NEO (**B**).

Performance Liquid Chromatography (Chiral-HPLC) results (Figure 2) showed that (*S*)-neoponcirin was obtained as a single epimer, with 98% of purity and the negative optical rotation value also confirmed that this corresponds to the 2*S* diasteroisomer [13].

### 2.2. Pharmacological Evaluations

#### 2.2.1. Antinociceptive Effect of NEO

**Figure 3:**
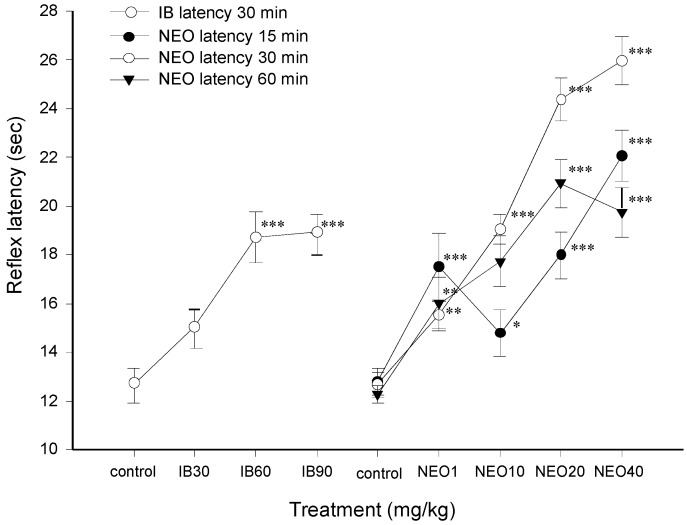
Effect antinociceptive of ibuprofen (IB) and NEO in the hot plate test.

The antinociceptive effect of NEO was evaluated in the hot plate thermal stimulation model, which has been widely used to evaluate analgesic effects mediated by central mechanisms [14]. As shown in Figure 3 ibuprofen (IB) was effective to prolonging the reflex latency response respect to the control group, similarly the i.p. administration of NEO at doses of 1, 10, 20 and 40 mg/kg produced a statistically significant increase in the reflex latency response, showing better effect at 30 min later of i.p. administration (−15 min: F_7,56_ = 7.335, *p* ≤ 0.001, −30 min: F_7,64_ = 10.924, *p* < 0.001, and −60 min: F_7,64_ = 5.534, *p* < 0.001). These results showed that NEO exerts a dose-dependent antinociceptive effect against thermal stimulus, this effect was better than that produced by IB; a potent non-opioid analgesic (NSAIDs) which has been reported to be effective in the hot plate test [15]. Thus, when comparing the actions of NEO with those of IB, it can be observed that NEO is clearly more potent than IB. 

All values are presented as means ± S.E.M, n= 8–10 animals for each group. Comparisons were made by using One Way Analysis of Variance; Administration latency 15 min: F (7, 56) = 7.335, *p* ≤ 0.001; Administration latency 30 min: F (7, 64) = 10.924, *p* < 0.001; Administration latency 60 min: F (7, 64) = 5.534, *p* < 0.001, followed by a multiple comparisons versus control group by Dunnett’s method; * *p* < 0.05, ** *p* < 0.1, *** *p* < 0.001.

**Figure 4:**
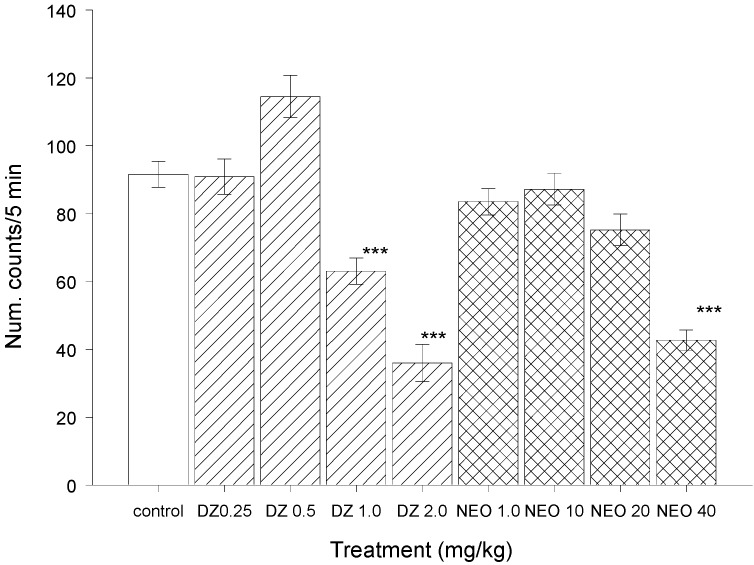
Effects of diazepam (DZ) and 2(*S*)-neoponcirin on locomotor activity.

Furthermore, the antinociceptive effects produced by NEO are not associated with any adverse effects since these doses did not induce hyper- or hypo-locomotion or any other visible motor disturbances, such as rigidity or tremor in the experimental animals when tested in the Open Field (Figure 4). Only at highest dose NEO (40 mg/kg) produced a decrease in the ambulatory activity of mice, which could interfere with its antinociceptive effect. The findings showed that NEO is an active priciple of *C. mexicanum*, and highlights its pharmacological potential for the development of new analgesics drugs.

Figure 4 shows the actions of NEO on the ambulatory activity of experimental animals. This natural molecule did not induce any change in the behavior of animals. Only at 40 mg/kg did NEO induce a decrease in the number of counts in the open field. In general a reduction in animals’ activity could be probably due to sedative actions. However, NEO did not show effects in the sleeping time test thus, this could be attributed to nonspecific effects.

All results are expressed as means ± SEM of 8–10 animals per group. Comparisons were made by using Kruskal-Wallis One Way Analysis of Variance on Ranks followed by the Mann-Whitney *U* test: * *p* < 0.05, ** *p* < 0.01 and *** *p* = 0.001. Kruskal-Wallis test data: H = 59.37, fd = 8, *p* ≤ 0.001; ^a^ control *vs.* DZ 0.25; T = 81.5, P = 0.65; ^b^ control *vs.* DZ 0.5; T = 94.5, *p* = 0.10; ^c^ control *vs.* DZ 1.0; T = 36.0, *p* ≤ 0.001; ^d^ control *vs.* DZ 2.0; T = 36, *p* ≤ 0.001; ^e^ control *vs.* NEO1.0; T = 78.5, *p* ≤ 0.36; ^f^ control *vs.* NEO10; T = 88.5, *p* ≤ 0.93; ^g^ control *vs.* NEO 20; T = 45.0, *p* ≤ 0.001; ^h^ control *vs.* NEO 40; T = 45.0, *p* ≤ 0.001.

#### 2.2.2. Test of Sodium Pentobarbital-induced Sleeping Time

NEO administration did not produce changes in neither the latency sedation nor the sleeping time induced by sodium pentobarbital. These results indicate that the NEO did not exert sedative effects (data no showed) at any of the doses tested. In this respect, it has been described that pentobarbital allosterically enhances the GABAergic neurotransmission by increasing the average open time of channel in a GABA-dependent fashion [16,17]. It is therefore possible that NEO is unable to modify this mechanism.

#### 2.2.3. Hole-Board Test

The hole-board test has been extensively used to detect anxiety-like effects produced in mice by drugs or active principles from medicinal plants [18]. Table 1 shows the effects of both DZ (0.5–4.0 mg/kg) and NEO (1–40 mg/kg) on the performance of mice in the hole-board test. In a similar manner, both treatments at low doses (NEO at 1 to 10 mg/kg and DZ at 0.5 mg/kg) significantly increased the head dipping number (H = 57.75, df = 8, *p* ≤ 0.001) and the rearing number (H = 61.73, df = 8, *p* ≤ 0.001) relative to the group control. Interestingly, in this test, both DZ and NEO at the highest doses tested had no effect. This lack of anxiolytic-like effect of both DZ and NEO at the dose of 4.0 mg/kg and 40 mg/kg respectively could be due to a sedative action, since at the same dose those drugs significantly decreased general activity as measured in the open field test. Thereby, NEO showed a pharmacological biphasic profile similar to that of DZ, a classic benzodiazepine, which at small doses causes anxiolytic effects while sedative effects are observed at increasing doses. In this regard, it is well known that benzodiazepines such as diazepam produce anxiolytic effects in humans and anxiolytic-like responses in most animal models through their interaction with the GABAergic system. These facts suggest that the GABAergic system could be involved in the anxiolytic effects produced by NEO.

**Table 1:** Effect of NEO on the performance of mice in the hole-board test.

**Treatment (mg/kg)**	**RN**	**HDN**
	Mean ± SEM	Mean ± SEM
Control	10.37 ± 0.80	26.12 ± 1.55
1.0	17.5 ± 1.74 **	32.33 ± 2.16 *
10.0	20.70 ± 0.98 ***	38.20 ± 3.33 **
20.0	5.0 ± 1.08 ^++^	24.77 ± 1.98
40.0	1 ± 0.50 ^+++^	6.8 ± 3.88 ^+++^
DZ 0.5	20.25 ± 1.66 ***	42.27 ± 1.55 **
DZ 1.0	13.44 ± 2.28	34.55 ± 3.12
DZ 2.0	9.66 ± 2.27	30.55 ± 3.61
DZ 4.0	3.08 ± 0.81 ^+++^	0.8 ± 0.44 ^+++^
	*H = 61.73*, *fd = 8(p ≤ 0.001 )*	*H = 57.75*, *fd = 8(p ≤ 0.001)*

All results are expressed as means ± SEM of 8–10 animals per group. Comparisons were made by using Kruskal-Wallis One Way Analysis of Variance on Ranks followed by the Mann-Whitney *U* test: * *p* ≤ 0.05, ** *p* ≤0.01 and *** *p* ≤ 0.001 (*, **, and *** symbol used to symbolize significant increases; +++ symbol used to symbolize significant decreases).

Benzodiazepines mainly exert their effects through interaction with the GABAergic system. The GABA_A_ receptors are the predominant receptors for GABAergic neurotransmission, when the endogenous GABA binds to GABAergic receptors at the GABA-binding site, the conductance to Cl^−^ ion increases in the neuronal membrane, which results in a hyperpolarization. This polarization in turn, leads to a reduced neuronal excitability [19]. The benzodiazepines are positive modulators of GABAergic neurotransmission, *i.e.*, GABA is required to produce the anxiolytic actions of benzodiazepines [20,21,22], whilst, GABAergic agonists such as muscimol interact directly with the GABA binding site to activate Cl^−^ ion conductance, and thus function even in the absence of GABA. In contrast agents that inhibit GABAergic neurotransmission are anxiogenic, for example, the GABA_A_ receptor antagonist picrotoxin, produces anxiogenic-like effects in mice. Picrotoxin is a selective non-competitive antagonist of GABA that specifically blocks the GABA_A_ receptor [16] thereby preventing the neuronal inhibitory effect of GABA. On the other hand, it is well known that many flavonoid compounds exert their anxiolytic and sedative effects through their direct or indirect actions on the GABAergic system [23,24]. Based on the hypothesis that GABAergic system could contribute to the anxiolytic actions of NEO, the co-administration of picrotoxin and MUS in combination with NEO was evaluated.

#### 2.2.4. Picrotoxin Blockade and Muscimol Synergism Experiments

**Figure 5:**
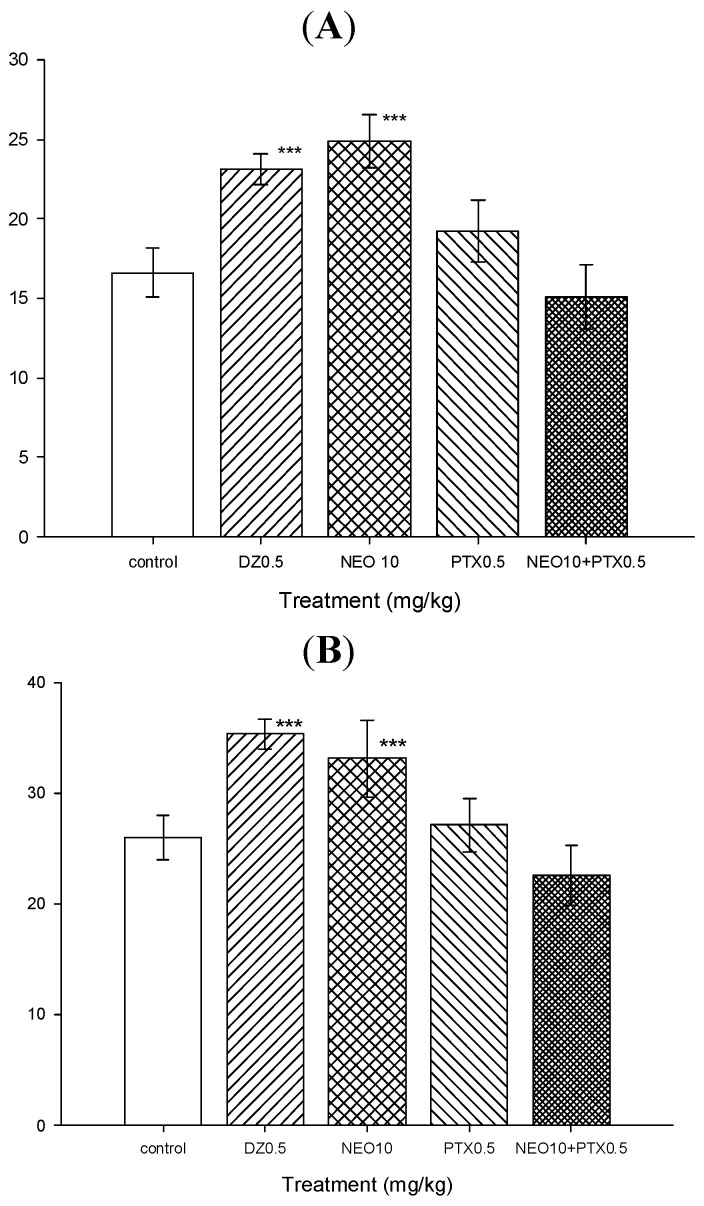
Effects of picrotoxin (PTX) on the anxiolytic-like effects of NEO in hole-board test. (**A**) Rearing number *vs.* treatment; (**B**) Head dipping number *vs.* treatment.

In order to explore possible participation of GABAergic system in NEO anxiolytic effects, the effects of picrotoxin and muscimol on the anxiolytic-like effect of NEO were analyzed. As it can be seen in Figure 5, NEO (10 mg/kg) significantly increased the rearing number (24.87 ± 1.69) and the head dipping number (34.12 ± 2.5) in respect to control group (rearing number = 16.62 ± 1.54 and head dipping number = 24 ± 2.0) in the hole-board test, when NEO was simultaneously administrated with PTX (0.5 mg/kg), the GABAergic antagonist picrotoxin was able to completely abolish the anxiolytic-like effect of NEO as the results of Mann-Whitney test showed no significant differences in rearing number, neither in head dipping number between vehicle-treated group and NEO (10 mg/kg) plus picrotoxin group (0.5 mg/kg) (rearing number; 15.20 ± 2.03; T = 81, n = 8, p = 0.19) and head dipping number (27.12 ± 2.4; T = 72, n = 8, p = 0.72) were observed (Figure 5). 

All results are expressed as means ± SEM of 8–10 animals per group. Comparisons were made by using Kruskal-Wallis One Way Analysis of Variance on Ranks followed by the Mann-Whitney *U* test: * *p* < 0.05, ** *p* < 0.01 and *** *p* = 0.001.

As shown by these results, the combination of PTX plus NEO was able to completely abolish the anxiolytic-like effects of NEO; this suggests that NEO could facilitate GABAergic transmission [25,26]. 

**Figure 6:**
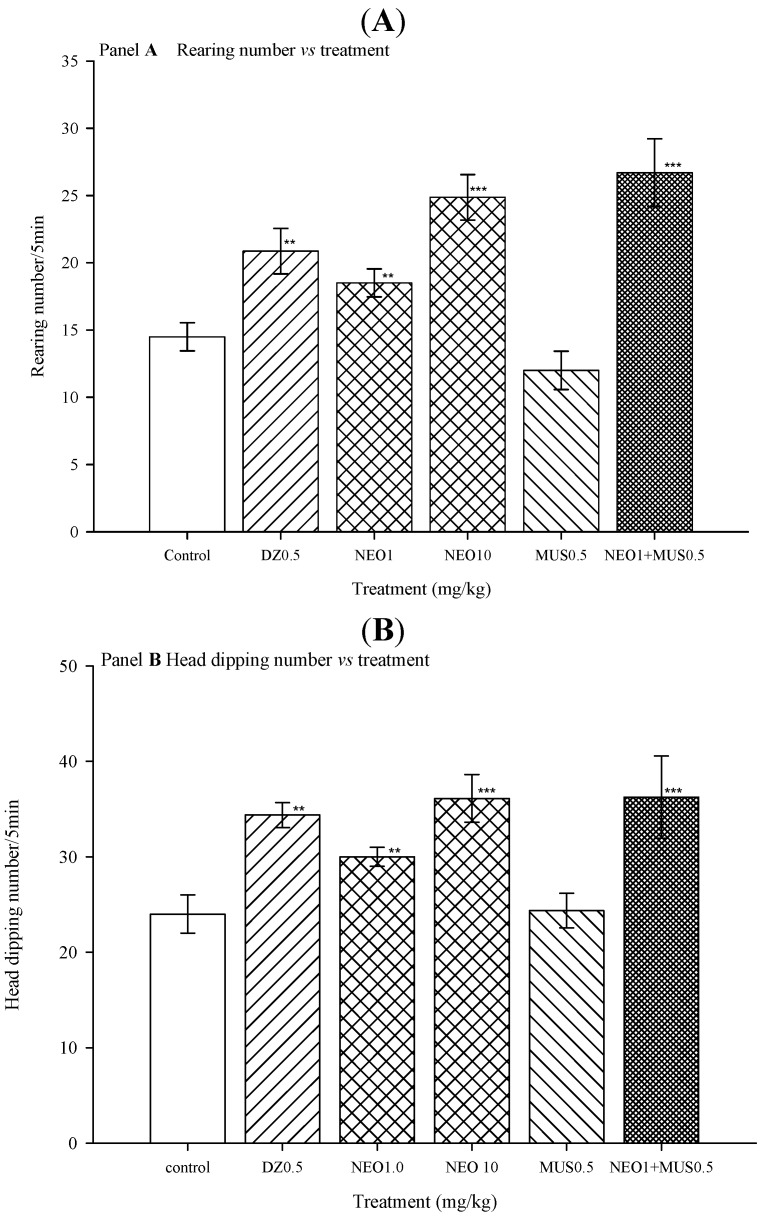
Effect of co-administration of muscimol (MUS) plus NEO in hole-board test. (**A**) Rearing number *vs.* treatment; (**B**) Head dipping number *vs.* treatment.

On the other hand, a sub-threshold dose of MUS, which is an specific agonist to the GABA_A_ receptor [16] was able to synergize with a non-effective dose of NEO (1 mg/kg) and produced anxiolytic-like effects in HBT, noted as an increase in both rearing and head dipping number when compared with muscimol-treatment alone and NEO-treatment (1 mg/kg) alone, this anxiolytic-like effect was similar to the one obtained with NEO at 10 mg/kg; (rearing number: H = 27.65, df = 4, *p* ≤ 0.001, head dipping number: H = 15.36, df = 4, *p* = 0.004) (Figure 6). The anxiolytic–like effects observed when the animals were simultaneously administered with MUS and NEO supports the idea that NEO could interact directly with the GABA binding site in a similar way to the agonist MUS.

All results are expressed as means ± SEM of 8–10 animals per group. Comparisons were made by using Kruskal-Wallis One Way Analysis of Variance on Ranks followed by the Mann-Whitney *U* test: * *p* < 0.05, ** *p* < 0.01 and *** *p* = 0.001.

These results are a good index of the GABAergic system participation on the anxiolytic actions of NEO; furthermore, this fact suggests that NEO could exert its anxiolytic-like effect through the interaction with the GABA_A_ receptors. However, future experiments could be conducted to determine the specific form of action. 

Our findings indicate that the molecular target of NEO to exert its actions is, at least partially, the central GABAergic system, producing an anxiolytic-like effect in mice. Nonetheless, NEO could also interact with other neurotransmitter systems, such as the serotoninergic, dopaminergic, noradrenergic, metabotropic systems of the GABA_B_ receptor or the opioid system that could contribute to the anxiolytic-like effects and even more to the antinociceptive effects of NEO. For example, it is well accepted that the opioids system plays a role in the anxiolytic actions of benzodiazepines, and they also modulate affective behaviors. Several studies have shown that opioid antagonists such as naloxone can block the anxiolytic responses to benzodiazepines [27,28], furthermore, it has been reported that the flavonol glucoside; gossypin activates opioid receptors, as it exhibits analgesic effects that are antagonized by the opioids antagonist naloxone [29,30]. However, further studies currently in progress will enable us to understand the mechanism of action underlying and its possible relationship with the NEO anxiolytic properties observed in this investigation. 

## 3. Experimental

### 3.1. General

The solvents used in this work were purchased from Aldrich (Aldrch Co., Toluca de Lerdo, Mexico). Chromatography was performed using an open column packed with 60 G F_254_ Merck silica gel at a ratio of 1:15 in respect to the extract. The chromatographic columns (CC) were monitored by thin layer chromatography TLC, and developed plates were visualized under a short and long-wave UV lamp and heating plates that were dipping in Ce_2_SO_4_/H_2_SO_4_, and/or NH_4_OH solutions. Melting points, were determined with a Fisher-Johns apparatus, and are uncorrected. Optical rotation was measured at 254 nm on a Perkin Elmer 341 digital polarimeter using a 100 mm cell and MeOH was used as solvent. ^1^H-NMR (400 MHz) and ^13^C-NMR (100 MHz) experiments were conducted on a Varian Mercury 400 MHz instrument spectra data using TMS as internal standard and DMSO-*d*_6_ (99.9% D) was used as solvent. Chemical shifts are reported in ppm with respect to TMS (tetramethysilane; δ = 0), and *J* are reported in Hz. The identity and purity of the isolated compound were confirmed by comparison of their physical and spectral properties (^1^H-NMR, ^13^C-NMR, and MS) with those reported in the literature, and through HPLC-ESI-MS analysis

### 3.2. Plant Material

Leaves of *Clinopodium mexicanum* Benth. Govaerts (Lamiaceae), were collected in Chilapa, Guerrero State, Mexico. The species was authenticated by botanist M. R. García Peña, and a voucher specimen was deposited in the Herbario del Instituto de Biología, Universidad Nacional Autónoma de México (MEXU No. 946030).

### 3.3. Isolation and Identification of 2S-Neoponcirin

Dried and finely ground *C. mexicanum* (100 g) was extracted successively with hexane (Hx), ethyl acetate (EtOAc), and methanol (MeOH). Evaporation of the solvents under vacuum yielded the respective extracts. The methanol extract was fractionated on a silica gel column; eluted with a hexane-ethyl acetate gradient (9:1→1:9) followed of ethyl acetate. From the fractions eluted with EtOAc: MeOH (1:1) of MeOH crude extract, a complex mixture of whitish solid was obtained by precipitation (2.56 g), The ^1^H-NMR data revealed the presence of neoponcirin, naringenin and hesperidin in this mixture. The mixture obtained was separated by CC using Sephadex LH 20 as phase solid and a mixture of ethanol: water (9:1) was employed as eluent, from this column 0.905 g of neoponcirin was obtained through re-crystallizations as an amorphous white solid with decomposition point 256–258 °C in a EtOAc:MeOH system. Optic rotation ${\left[\alpha \right]}_{D}^{25}$ = −45° (0.002 g/mL in MeOH).

In order to determine the purity of NEO, an analytical method was developed using high performance liquid chromatography (HPLC). The analysis was carried out with an Agilent model 1264 analytical HPLC. Chromatographic conditions were as follows: a chiral column: Chiracel OD-RH, the mobile phase was acetonitrile-water system (ACN:H_2_O) with a program ramp 20:80 to 80:20 v/v at 0 min to 20 min, flow-rate was 1 mL/min and the injection volume was 5 μL of concentrate solution of NEO in water. The temperature was established and set at 25 °C, and the detection wavelength was 254 nm. The purity of NEO was quantified by its HPLC peak as % relative area. The HPLC peak area was not corrected for response factors. NEO was obtained with a 98% of purity, with an RT = 6.04 min, and according to its optical rotation value, this corresponds to the 2*S* isomer.

Additionally, a positive ion mass spectrum of the column eluted was recorded in electrospray mass spectrometry (ESI-MS) in the range of m/z 100–1,000 amu with a scan cycle time of 2 s. ESI-IT source conditions were adjusted as follows: drying N_2_ at 10 L/min, capillarity temperature = 350 °C, spray voltage = 40.0 volts, auxiliary gas pressure = 30 psi [9].

The NMR data are as follows: ^1^H-NMR δ 12.0 (1H, s broad, int-D_2_O, C_5_-OH), 7.45 (2H, d, *J* = 8.7 Hz, H-2′, 6′), 6.98 (2H, d, *J* = 8.7, H-3′, 5′), 6.12 (2H, d, *J* = 2.8 Hz, H-6, 8), 5.57 (1H, dd, *J* = 2.8, 12.6 Hz, H-2), 4.98 (1H, d, *J* = 7.5 glc H-1′′), 4.52 (1H, s broad, rha H-1′′′), 3.80 (1H, H-6a′′), 3.76 (3H, s, OCH_3_), 3.65 (1H, H-2′′′), 3.54 (1H, H-5′′), 3.44 (1H, H-3′′′), 3.43 (1H, H-6b′′), 3.39 (1H, H-5′′′), 3.28 (1H, H3′′), 3.23 (1H, H-2′′), 3.16 (1H, H-4′′′), 3.14 (1H, H-4′′), 3.36 (1H, dd, *J* = 12.6, 17.1 Hz, H-3a), 2.77 (1H, dd, *J* = 2.7, 17.1 Hz, H-3b), 1.09 (3H, d, *J* = 6.23 Hz, H6′′′). ^13^C-NMR δ 197 (C-4), 165 (C-7), 162.9 (C-5), 162.6 (C-9), 159.5 (C-4′), 130 (C-1′), 128 (C-2′, 6′), 113 (C-3′, 5′), 103.3 (C-10), 100.5 (C-1′′, C anomeric-glc ), 99.4 (C-1′′′, C anomeric-rha ), 96. (C-6), 95.5 (C-8), 78.1 (C-2), 76.2 (C-5′′), 75.6 (C-3′′), 72.8 (C-2′′), 70.7 (C-4′′′), 70.1 (C-3′′′),69.6 (C-2′′′) 69.5 (C-4′′), 68.3 (C-5′′′), 66 (C-6′′), 55 (OCH_3_), 41.7 (C-3), 17.9 (C-6′′′). 

### 3.4. Pharmacological Evaluations

#### 3.4.1. Animals

Adult male Swiss Webster mice (20–30 g) were obtained from the vivarium at the Instituto Nacional de Psiquiatría Ramón de la Fuente Muñiz (México, D.F., Mexico). All animals were housed eight per cage in a temperature-controlled (20–21 °C) room under inverted light:dark conditions (12:12 h, lights on at 22:00 h). All behavioral evaluations were performed between 10:00 and 14:00 h. Animals had *ad libitum* access to Purina rodent chow and water. Animals were handled in agreement to the general principles of laboratory animal care (publication # 85–23, revised in 1985) [31] and the ‘Norma Oficial Mexicana’ (NOM-062-ZOO-1999) further, the experimental protocol was approved by the local ethical committee (NC093620.0). All the experimental sessions were videotaped and analyzed by an observer unaware of the treatment conditions. For habituation, animals received a daily i.p. injection of saline solution (0.1 mL/10 g) for five days before treatments were initiated. Diazepam (intraperitoneal route; i.p.) was used as a reference drug in both the sedative and antianxiety tests, and ibuprofen (IB; via i.p.) was used as control group in the antithermonociceptive test. Doses for the NEO were determined in previous pilot studies carried out in our laboratory.

#### 3.4.2. Drug Preparation and Dosage

All drugs in this study were intraperitoneally (i.p.) injected in a total volume of 10.0 mL/kg body weight. Sodium pentobarbital (SP, Sigma Chemical Co., St. Louis, MO, USA) was dissolved in an isotonic solution (0.9% NaCl), diazepam (DZ, Hoffmann-La Roche, Mexico City, Mexico) and ibuprofen (IB, Aldrich-Sigma, Mexico City, Mexico), muscimol (MUS, Sigma Chemical Co., St. Louis, MO, USA), picrotoxin (PTX, Sigma Chemical Co., St. Louis, MO, USA), were dissolved in 1.0% propylene glycol (Pg 600). Control animals received the same volume of the vehicle (isotonic saline, 0.9% NaCl). The pharmacological assays were performed with aqueous dilutions of the NEO containing 1%–2% Tween 80. Doses are expressed as milligrams of NEO per kilogram of body weight for each mouse.

#### 3.4.3. Hot Plate Test

A total of twenty-two groups of eight mice each were employed in the hot plate test. Twelve independent groups were administered NEO intraperitoneally at doses oft 1.0, 10, 20 or 40 mg/kg, and were evaluated in three independent experiments carried out at 15, 30 and 60 min (latency time) before the beginning of the test. Another group administrated only with saline solution was used as control. These experiments were carried out in order to establish the administration latency of NEO. In the same form, three independent groups of animals were treated with ibuprofen at 30, 60 and 90.0 mg/kg doses to 30 min before beginning the test. These groups served as positive controls. 

In this test, each mouse was introduced into a glass cylinder (20 cm in diameter and 25 cm in height) placed at the center of a metal plate (Uge baseline, model DS 37) adjusted to 53 ± 0.5 °C. Within several seconds, the animals displayed specific responses evoked by the thermal stimulation, including the flexor antialgesic reflex behavior. If the mouse did not respond within 50 seconds, the test was terminated, and the mouse was immediately removed from the hot plate to avoid tissue damage and returned to its home cage. Animals were tested one at a time and were not habituated to the apparatus prior to testing. Each animal was tested only once [9,15].

#### 3.4.4. Open Field Test

NEO was administered at doses of 1, 10, 20, and 40 mg/kg (i.p.) to four independent groups of mice and spontaneous locomotor activity was measured 30 min after of administration. The Open Field Test involves an apparatus made of an opaque-Plexiglas box (40 cm × 30 cm × 20 cm) with the floor divided into 12 equal squares. Each animal was gently placed in a corner of the apparatus and its behavior was videotaped during a 5 min session. A blind observer registered the number of times the animal entered each square (counts per 5 min) A count is considered when the animal totally crosses from a square to the next. A change in the number of counts respect to control group is considered as an alteration of locomotor activity [25]. 

#### 3.4.5. Test of Sodium Pentobarbital-Induced Sleeping Time

The sedative and hypnotic effects of NEO in combination with sodium pentobarbital (SP) were evaluated. For this purpose, a total of 64 mice were divided into eight groups (n = 8). Five groups received NEO (1.0, 10, 20, and 40.0 mg/kg i.p., respectively) 30 min before the administration of SP (42 mg/kg, i.p.). Two other groups received DZ (0.5 and 1.0 mg/kg, i.p., respectively) 30 min before the administration of SP (42 mg/kg, i.p.); these two groups served as positive controls. An independent group was injected with the vehicle 30 min before the i.p. administration of the 42 mg/kg dose of SP, and this group served as a negative control. Each mouse was placed on a warm table and carefully observed for the onset of uncoordinated movements corresponding to the sedative phase of the test. Loss of the righting reflex related to the hypnosis phase and the duration of sleep were also observed. The time elapsed between the loss and recovery of the righting reflex was considered as the sleeping time [32].

#### 3.4.6. Hole-Board Test

The hole-board set-up apparatus is a wooden box of 60 cm × 30 cm × 30 cm with four equidistant holes (2 cm diameter) on the floor. NEO was administered at increasing doses (1.0, 10, 20 and 40 mg/kg, i.p.) to independents groups of eight mice each. After 30 min, each mouse was placed in the center of the hole-board and the number of head-dips into the holes and the number of rears was recorded over a 5 min period. A head-dip was registered if a mouse put its head in a hole at least up to the eye level; repeated dips into the same hole were not counted unless these were separated by locomotion and rearing is scored when mice stand up themselves on the hind legs and the fore-paws rest on a partition wall.

Four independent groups of eight animals received DZ (0.5, 1, 2, and 4 mg/kg) 30 min before the test was conducted and these groups served as a reference. One group receiving only the vehicle served as a control. An increase in both the number of head-dips and the number of rears compared to the controls were considered to indicate an anxiolytic effect, while a decrease of those was considered as a sedative effect After each trial, the floor of the apparatus was carefully cleaned to remove traces of previous paths [9].

#### 3.4.7. Picrotoxin Blockade Experiments

To explore the role of GABA system in CNS anxiolytic-like effects of NEO the pre-treatment with picrotoxin (PTX) was employed to attempt blocking the pharmacological effect of NEO in the hole-board test. NEO to 10 mg/kg dose was selected for this assay. PTX (0.5 mg/kg) and NEO were administered 30 min and 25 min, respectively, prior to testing.

#### 3.4.8. Muscimol Synergism Experiments

To explore the possible synergistic effect of muscimol (MUS) on anxiolytic-like effect of NEO, the experimental animals were co-administered with NEO (1 mg/kg) and a sub-threshold dose of MUS (0.5 mg/kg), 30 min before set on test. Doses selected for these experimental series were chosen based on the results obtained in the first hole-board experiment.

### 3.5. Statistical Analysis

When the differences between treated and control groups data did not meet normality or variance equality criteria, were analyzed using the non-parametric tests of Kruskal-Wallis analysis of variance on ranks (* *p* < 0.05, ** *p* < 0.01, and *** *p* < 0.001), followed by the Mann-Whitney Rank Sum test. Whereas, data did meet normality or variance equality criteria, the significance was assessed by a one-way ANOVA, followed by Dunnett’s multiple comparisons test. 

## 4. Conclusions

The findings of the present work demonstrated that the (*2S*)-5-hydroxy-4′methoxy flavanone-7-*O*-{*β*-glucopyranosyl-(1→6)-*β*-rhamnoside} isomer of neoponcirin produces antinociceptive and anxiolytic-like effects in mice. Our findings contributed at least partially to identify the mechanism subjacent to anxiolytic-like effect produced for neoponcirin, which involves the participation of GABA_A_ receptor. On the basis of the present results it may be suggested that 2(*S*)-neoponcirin might be of interest as an anxiolytic drug for the treatment of anxiety and for treatment of pain. Additionally, these observations support the idea that to *C. mexicanum* possess analgesic and sedative properties may be due to the presence of 2(*S*)-neoponcirin. Finally, the present paper contributes to our knowledge about active principles from medicinal plants used in Mexican traditional medicine. It shows its potential for the development of new and better analgesic and anxiolytic drugs.
